# Distribution and Relative Abundance of Bean Leaf Beetles (*Ootheca* spp.) (Insecta: Coleoptera: Chrysomelidae) in Uganda

**DOI:** 10.3390/insects12111048

**Published:** 2021-11-22

**Authors:** Charles Halerimana, Samuel Kyamanywa, Samuel Olaboro, Pamela Paparu, Stanley T. Nkalubo, John Colvin, Robert A. Cheke, Thomas Wagner, Susan E. Seal, Darren J. Kriticos, Michael H. Otim

**Affiliations:** 1 Department of Agricultural Production, College of Agricultural and Environmental Sciences, Makerere University, Kampala P.O. Box 7062, Uganda; chahalerimana@gmail.com (C.H.); skyamanywa@gmail.com (S.K.); 2 National Coffee Research Institute, Mukono P.O. Box 185, Uganda; 3 National Crops Resources Research Institute, Kampala P.O. Box 7084, Uganda; olasamuel94@gmail.com (S.O.); pamela.paparu@gmail.com (P.P.); tamusange@gmail.com (S.T.N.); 4 Natural Resources Institute, University of Greenwich at Medway, Central Avenue, Chatham Maritime, Kent ME4 4TB, UK; J.Colvin@greenwich.ac.uk (J.C.); R.A.Cheke@greenwich.ac.uk (R.A.C.); S.E.Seal@greenwich.ac.uk (S.E.S.); 5 Institut für Integrierte Naturwissenschaften—Biologie, Universität Koblenz-Landau, Universitätstrasse 1, 56070 Koblenz, Germany; thwagner@uni-koblenz.de; 6 Commonwealth Scientific and Industrial Research Organization GPO 1700, Canberra 2601, Australia; Darren.Kriticos@csiro.au; 7 School of Biology, University of Queensland, Brisbane 4072, Australia

**Keywords:** abundance, agro-ecological zone, common bean, *Ootheca* spp., damage

## Abstract

**Simple Summary:**

Bean leaf beetles (*Ootheca* spp.) (Insecta: Coleoptera: Chrysomelidae) were originally thought to be minor pests of the common bean in Uganda, with only reports coming from the north. The beetles have already expanded to other locations, prompting farmers to postpone bean planting in order to avoid their damaging effects. The species that do exist in Uganda, however, are poorly documented. Furthermore, little is known about the factors that influence bean leaf beetle population dynamics and dispersion across the country. We conducted surveys to determine the species and relative abundance of bean leaf beetles, as well as the factors that control their population dynamics. We recovered 12 genera of leaf beetles on common bean from the sampled agro-ecological zones in Uganda. Only three species belong to the genus *Ootheca* (*O. mutabilis*, *O. proteus* and *O. orientalis*) in the surveyed locations of Uganda. *Ootheca mutabilis* is the most common of the three species, accounting for 70% of the total. The most infested agro-ecological zone is the Northern Moist Farmlands, whereas the least infested is the Southwestern Highlands. Our findings provide a foundation for assessing the importance of *Ootheca* species as common bean pests in Uganda.

**Abstract:**

Bean leaf beetles (*Ootheca* spp.) (Insecta: Coleoptera: Chrysomelidae) are one of Africa’s most destructive pests of common bean and other leguminous crops. The beetles are widely distributed in Africa where they are estimated to cause annual crop yield losses of 116,400 tons of crop yields in sub-Saharan Africa. Despite their importance, little is known about the distribution, relative abundance and damage caused by bean leaf beetles in Uganda. As a result, the development of effective management methods has been hampered. We conducted surveys in six key Ugandan agro-ecological zones to determine the species distribution and relative abundance of bean leaf beetles. Findings indicate that leaf beetles belonging to 12 genera are present, including members of the genera *Afrophthalma* Medvedev, 1980, *Buphonella* Jacoby, 1903, *Chrysochrus* Chevrolat in Dejean, 1836, *Diacantha* Dejean, 1845, *Exosoma* Jacoby, 1903, *Lamprocopa* Hincks, 1949, *Lema* Fabricius, 1798, *Nisotra* Baly, 1864, *Neobarombiella* Bolz and Wagner, 2012, *Ootheca* Dejean, 1935, *Parasbecesta* Laboissière, 1940, and *Plagiodera* Dejean, 1835. We identified only three species belonging to the genus *Ootheca*: *O. mutabilis*, *O. proteus*, and *O. orientalis*. Seventy percent of all the beetles collected were *O. mutabilis* and these were present in all agro-ecological zones studied. The Northern Moist Farmlands (21.9%), West Nile Farmlands (12.9%), Central Wooded Savanna (4.4%) and Southern and Eastern Lake Kyoga Basin (1.4%) were the only agro-ecological zones where *O. proteus* was found. Only one specimen of *O. orientalis* was found at a single site in the Central Wooded Savanna. The Northern Moist Farmlands had a significantly (*p* < 0.05) higher bean leaf beetle density than the West Nile Farmlands and Southwestern Highlands. Similarly, the Northern Moist Farmlands had the highest beetle foliar damage per plant (1.15 ± 0.05), while the Southwestern Highlands had the lowest (0.03 ± 0.02). We provide the first information on *Ootheca* species distribution, abundance and damage in Uganda. Our findings provide a foundation for assessing the importance of *Ootheca* spp. as common bean pests in Uganda.

## 1. Introduction

In Eastern Africa, the common bean (*Phaseolus vulgaris* L.) plays a critical role in human nutrition and income [1]. The crop is widely consumed in Uganda and provides a significant source of income for the majority of the country’s rural population [2]. However, yields have remained low, averaging 1.5 t ha^−1^ [3], compared to the potential of 2.5 t ha^−1^ for bush bean varieties and 3.5 t ha^−1^ for climbing types [4]. The yield disparities are attributed to many production constraints, including bean leaf beetles. Bean leaf beetles (*Ootheca* spp.) (Insecta: Coleoptera: Chrysomelidae) are endemic to mainland Africa and have become well-known as pests of common bean (*P. vulgaris*) and other leguminous crops such as cowpea [*Vigna unguiculata* (L.) Walp.], soybean [*Glycine max* (L.) Merr.], cucumber (*Cucumis sativus* L.), Sesbania [*Sesbania sesban* (L.) Merr.] and Bambara groundnut [*Vigna subterranean* (L.) Verdc.] [5,6,7]. *Ootheca* spp. have been described as common bean and cowpea pests in Uganda’s northern and eastern regions [8,9]. Bean leaf beetles skeletonize leaves and consume lateral roots, floral components, and immature pods [10]. In Tanzania, bean leaf beetles were responsible for bean yield losses of 18%–31% [11]. *Ootheca* species are frequent bean pests that force farmers to abandon growing the crop following the first seasonal rains in northern Uganda [9] and cause grain yield losses ranging from 5.8% to 48.9% [12].

According to a recent study of the genus *Ootheca*, there are 13 species occurring in Africa [7], eight of which have been collected in Uganda since 1957. The distribution of *Ootheca* spp. was previously documented to be influenced by altitude [7,13], host plants, growing season and cropping systems [14]. In Uganda, there is a scarcity of data on the current distribution, abundance and damage of *Ootheca* spp., limiting the development of management practices to combat them. The ecology of bean leaf beetles is critical for designing a successful pest management strategy.

We used field surveys to determine the species composition of bean leaf beetles, their distribution and relative abundance, associated damage, and factors influencing their population dynamics in this work.

## 2. Materials and Methods

### 2.1. Study Areas

**Figure 1:**
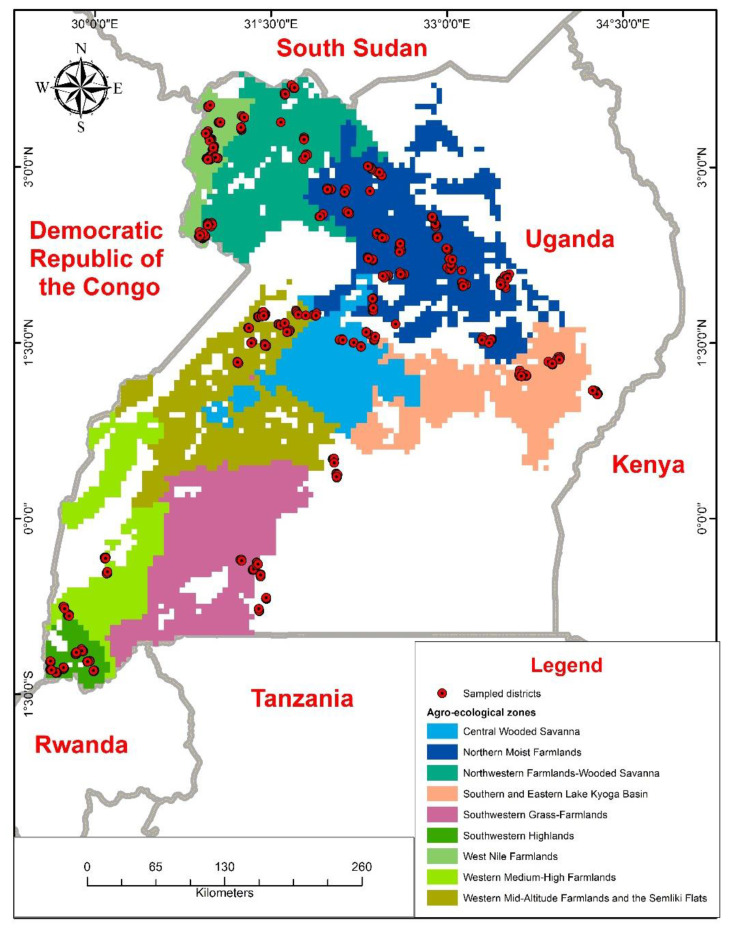
Map of Uganda showing locations where surveys were conducted in different agro-ecological zones.

As categorized in [15], our study was carried out in six main bean production agro-ecologies: Northern Moist Farmlands, West Nile Farmlands, Central Wooded Savanna, the Southern and Eastern Lake Kyoga Basin, the Southwestern Highlands, and the Western Mid-Altitude Farmlands and the Semliki Flats (Figure 1). The climatic/weather features and altitude varied [15]. The Southwestern Highlands, at an elevation of 2112 m above sea level (asl), are the highest in terms of altitude [15]. Western Mid-Altitude Farmlands and the Semliki Flats are next, being 1198 m asl, followed by Central Wooded Savanna with 1089 m asl, Southern and Eastern Lake Kyoga Basin with 1075 m asl, Northern Moist Farmlands with 1024 m asl, and West Nile Farmlands with 732 m asl. Table S1 shows the weather variables of the selected agro-ecological zones during the sampling period. The agro-ecologies were chosen based on bean production levels and geographical representation. While the target was the above listed six agro-ecological zones, the lack of bean crops in some districts of the targeted agro-ecologies led to sampling in other districts in the neighboring agro-ecological zone.

### 2.2. Assessment of Bean Leaf Beetle Abundance and Damage

**Figure 2:**
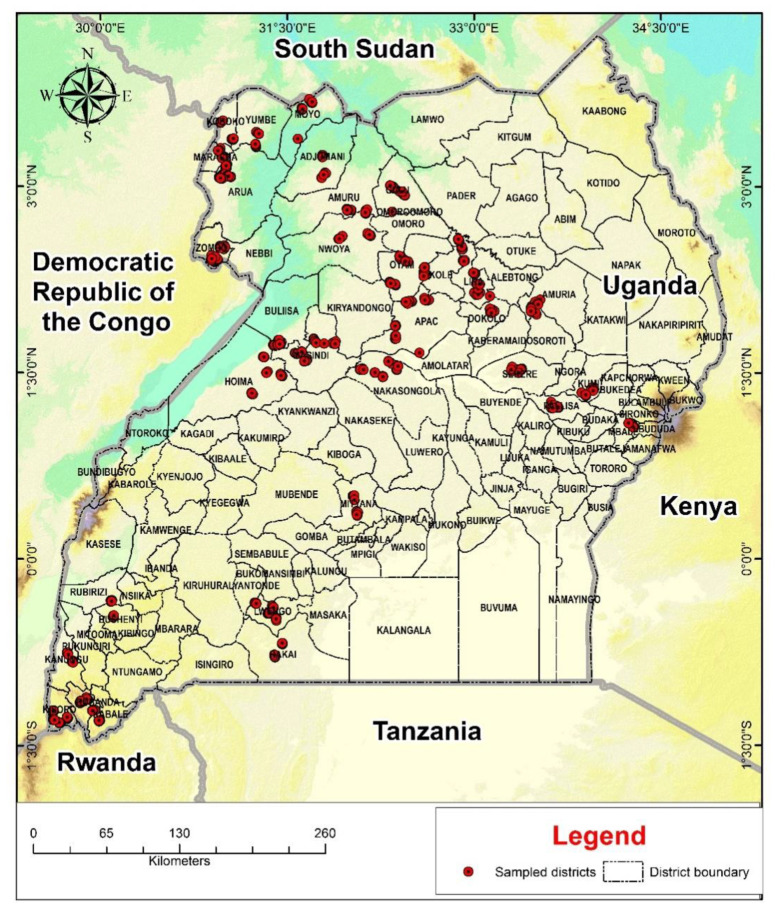
Map of Uganda showing the districts sampled during the study.

Field surveys were carried out in 2016 during the first (March to June) and second (September to November) rainy seasons and, in 2017, during the first (March to June) rainy season. During the 2016 and 2017 first rains, each agro-ecological zone was visited once while two of the six agro-ecological zones were visited twice during the 2016 second rains. Two trips were undertaken to the Northern Moist Farmlands, where beans are planted continuously from the first to the second seasonal rains, and the West Nile Farmlands, where there is only one long rainy season per year. The first and sole visit took place one month after the rains began, while the second visit took place one month later. From each agro-ecological zone, three districts were selected per visit. The districts chosen during the first visit or season were not always the same as those chosen during the second visit or season. This was intended to gather data on bean leaf beetles from a larger area inside an agro-ecological zone. Table 1 and Figure 2 show the districts sampled during the study period. Two subcounties were chosen from each district, from which ten fields were chosen. The furthest sampling field was chosen, as were the crops cultivated (bean sole crop or bean intercrop) and the stage of the bean crop. Bean leaf beetle populations were determined by direct counts on 20 plants from each field chosen from three one-square meter quadrats, with seven plants chosen from two quadrats and six plants chosen from the third. The undersides of the leaves were examined and any beetles that were discovered were recorded. Foliar damage caused by bean leaf beetles was evaluated using a damage rating scale of 0 to 5, where 0 = no defoliation, 1 = 1%–5% defoliation, 2 = 6%–25% defoliation, 3 = 26%–50% defoliation, 4 = 51%–75% defoliation, and 5 = 76%–100% defoliation [11]. Bean growth stage, cropping system, host plant density, cropping history, insecticide use history and sampling time (hour) were all recorded. Sampling time was categorized as morning hours (8 to 11 a.m.), afternoon hours (12 to 3 p.m.) and evening hours (4 to 5 p.m.). The vegetative and reproductive phases of bean growth were considered. V1 (completely unfurled leaves at the primary leaf node), V2 (first node above primary leaf node), V3 (three nodes on the main stem including the primary leaf node), V4 (four nodes on the main stem) and V5 (five nodes on the main stem) were the vegetative phases. R1 (one blossom open at any node), R2 (pods 1.27 cm long at first blossom position), R3 (pods 2.54 cm long at first blossom position), R4 (pods 7.62 cm long, seeds not discernible), R5 (pods 7–10 cm, seed discernible) and R6 (seeds at least 0.62 cm over long axis) were the reproductive phases [16]. Cropping systems were monocrop or intercrop, host plant density was high or low (low plant density was less than 15 plants m^−2^ and high plant density was greater than 15 plants m^−2^), cropping history was either host plant or non-host planted in the previous season and insecticide use history was whether chemical insecticides had been used in the field or not.

### 2.3. Identification of Bean Leaf Beetle Species

Collected adult bean leaf beetle specimens were preserved at the National Crops Resources Research Institute, Namulonge in 2 mL microcentrifuge tubes containing absolute ethanol before being sent to the Institute of Integrated Sciences of the University of Koblenz–Landau for identification. During storage, ethanol was replaced every two weeks for a month to ensure that the bean leaf beetle’s characteristics were not distorted. Bean leaf beetles were identified at the University of Koblenz-Landau, using protocols outlined in [7].

### 2.4. Data Analysis

Bean leaf beetle counts and foliar damage data from various agro-ecological zones and seasons were subjected to analysis of variance using a linear model function in R [17]. The three sampling time categories were also subjected to analysis of variance. When significant differences were found, the Tukey test was used to separate the means (*p* = 0.05). Aside from agro-ecological zones, the analysis included cropping system, host plant density, cropping history and insecticide usage history, all of which had two levels. The differences between the means for the two levels of each parameter were determined using a student’s *t*-test.

## 3. Results

### 3.1. Bean Leaf Beetle Species Composition in Uganda

**Figure 3:**
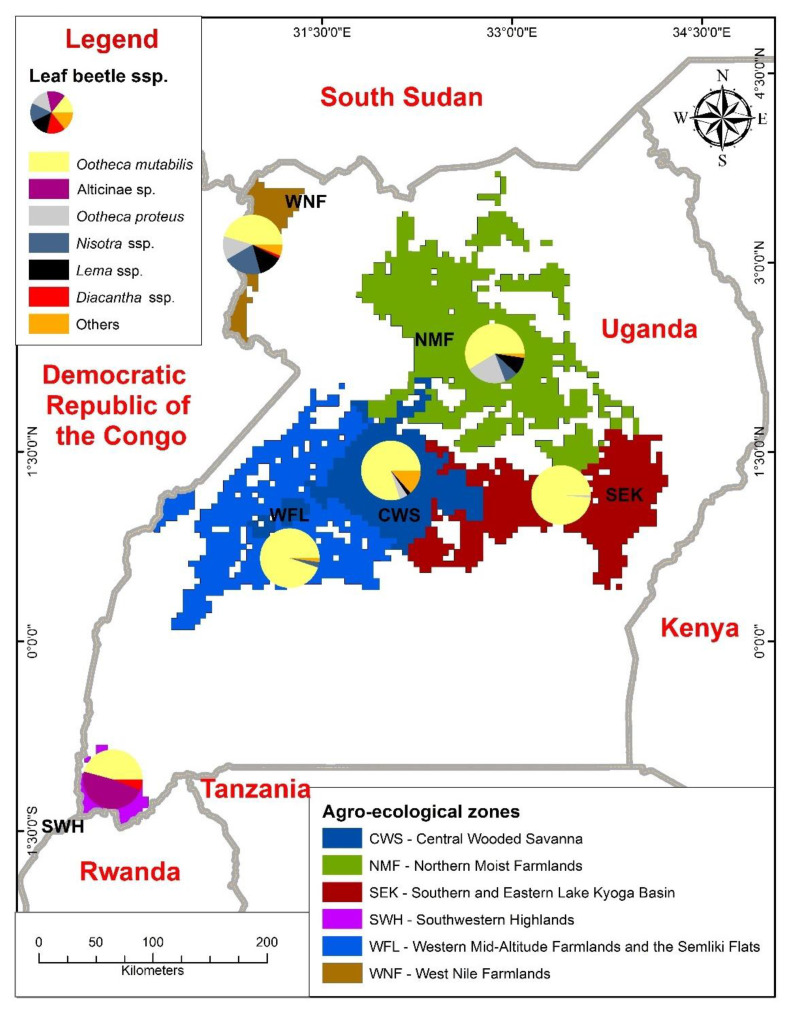
Composition of bean leaf beetles in different agro-ecological zones in Uganda.

There were 12 genera of bean leaf beetles from the sampled agro-ecological zones in Uganda. These included *Afrophthalma* Medvedev, 1980, *Buphonella* Jacoby, 1903, *Chrysochrus* Chevrolat in Dejean, 1836, *Diacantha* Dejean, 1845, *Exosoma* Jacoby, 1903, *Lamprocopa* Hincks, 1949, *Lema* Fabricius, 1798, *Nisotra* Baly, 1864, *Neobarombiella* Bolz and Wagner, 2012, *Ootheca* Dejean, 1935, *Parasbecesta* Laboissière, 1940, and *Plagiodera* Dejean, 1835. There were also leaf beetles belonging to subfamily Alticinae that were not identified further. Only three species of *Ootheca* were found in our study: *Ootheca mutabilis* Sahlberg, 1829, *O. proteus* Chapuis, 1879 and *O. orientalis* Weise, 1900. *Ootheca mutabilis* was the most common of the three species, found in all agro-ecologies, whereas *O. proteus* was found in only four (Table 2, Figure 3). Only one *O. orientalis* specimen was recovered (from the Central Wooded Savanna agro-ecological zone). Table S2 shows the breakdown of genera or species classified as others in Table 2.

### 3.2. Incidence of Bean Leaf Beetles and Foliar Damage across Agro-Ecological Zones

**Figure 4:**
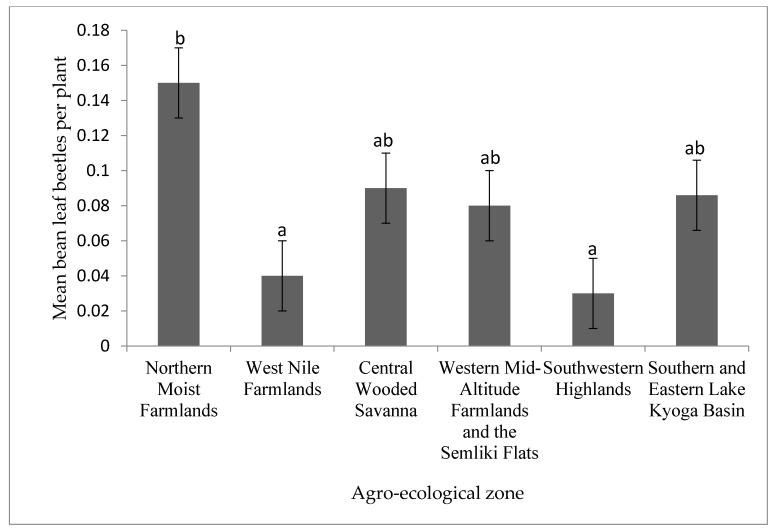
Incidence of bean leaf beetles in different agro-ecologies in Uganda. Bars bearing the same letter or letter combination are not significantly different at *p* = 0.05.

The density of bean leaf beetles was significantly (*p* < 0.001) different across the six agro-ecologies examined (Figure 4). Bean leaf beetle density was highest in the Northern Moist Farmlands, and lowest in the southwestern Highlands and West Nile Farmlands.

**Figure 5:**
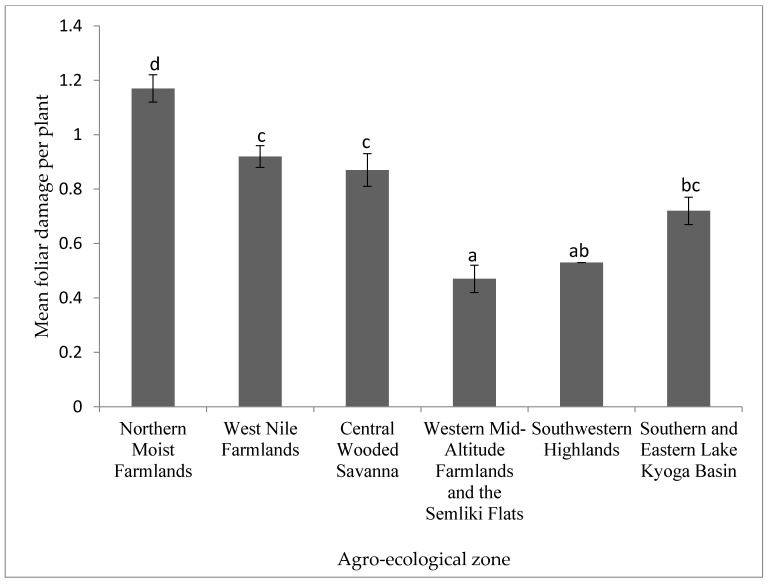
Foliar damage due to bean leaf beetles in different agro-ecological zones in Uganda. Bars bearing the same letter or letter combination are not significantly different at *p* = 0.05.

Foliar damage was also significantly (*p* < 0.001) different between agro-ecologies (Figure 5). Northern Moist Farmlands experienced the most foliar damage, whereas Western Mid-Altitude Farmlands and the Semliki Flats experienced the least.

### 3.3. Incidence of Bean Leaf Beetles and Foliar Damage in Different Seasons

**Figure 6:**
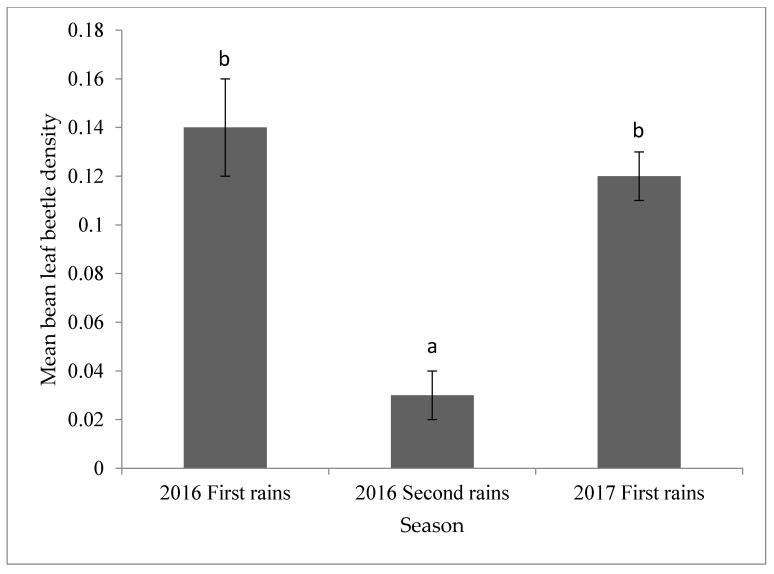
Incidence of bean leaf beetles across seasons in Uganda. Bars bearing the same letter are not significantly different at *p* = 0.05.

Bean leaf beetle population density varied significantly (*p* < 0.05) between growing seasons (Figure 6). The highest was in the first rainy season (March to June 2016 and March to June 2017), while the lowest was in the second rainy season (September to November 2016). There was no significant difference in foliar damage between the two seasons (Table S3).

### 3.4. Influence of Bean Growth Stages on Beetle Population Density and Foliar Damage

**Figure 7:**
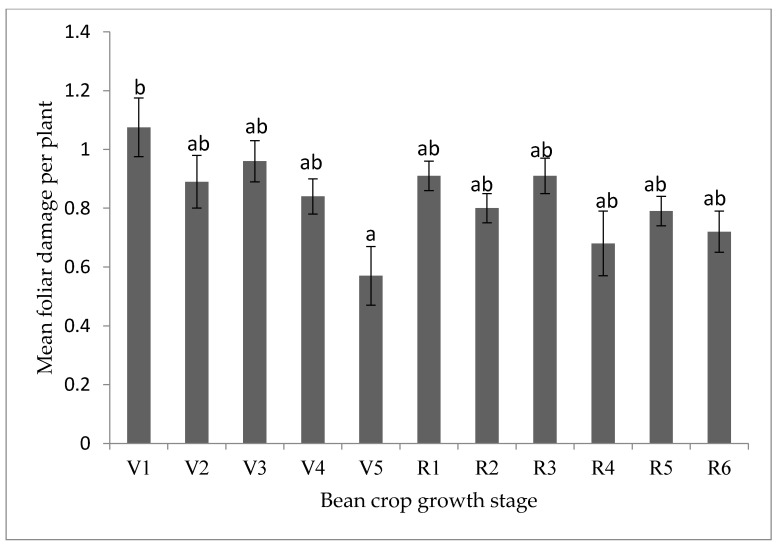
Influence of bean crop growth stage and reproductive phase on foliar damage due to bean leaf beetles in Uganda. Bars bearing the same letter or letter combination are not significantly different at *p* = 0.05. Key to bean crop growth stages: V1 (completely unfurled leaves at the primary leaf node), V2 (first node above primary leaf node), V3 (three nodes on the main stem including the primary leaf node), V4 (four nodes on the main stem) and V5 (five nodes on the main stem). Reproductive phases were R1 (one blossom open at any node), R2 (pods 1.27 cm long at first blossom position), R3 (pods 2.54 cm long at first blossom position), R4 (pods 7.62 cm long, seeds not discernible), R5 (pods 7–10 cm, seed discernible) and R6 (seeds at least 0.62 cm over long axis).

Bean leaf beetle density did not differ significantly (*p* > 0.05) among bean growth stages (Table S4). However, there were significant (*p* < 0.05) differences in foliar damage between bean growth stages (Figure 7). The highest foliar damage was recorded at the V1 stage while the lowest was recorded at the V5 stage.

### 3.5. Incidence of Bean Leaf Beetles and Foliar Damage under Different Cropping Systems

**Figure 8:**
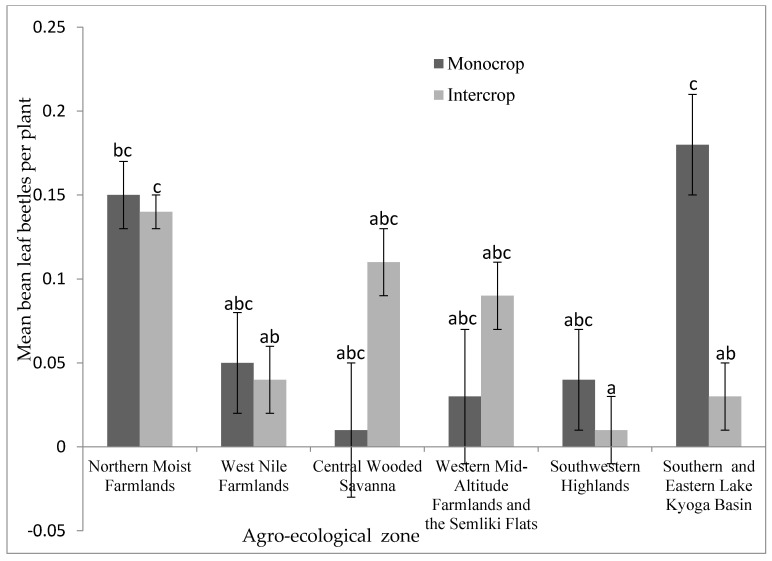
Incidence of bean leaf beetles under different cropping systems in different agro-ecological zones in Uganda. Bars bearing the same letter or letter combinations are not significantly different at *p* = 0.05.

The population of bean leaf beetles was significantly (*p* < 0.05) higher in the monocrop than in the intercrop only in the Southern and Eastern Lake Kyoga Basin (Figure 8). The population density did not differ significantly between monocrop and intercrop in the other five agro-ecological zones. Furthermore, the cropping system had no significant impact on foliar damage in all agro-ecological zones (Table S5).

In all agro-ecological zones, results from host plant density (Table S6), time of sampling (Table S7), cropping history (Table S8), and insecticide usage history (Table S9) for both bean leaf beetle density and foliar damage were not significant (*p* > 0.05).

## 4. Discussion

Our research is the first to comprehensively document the presence of *Ootheca* spp. in Ugandan bean agro-ecologies. *Ootheca mutabilis* occurred in all agro-ecologies studied, while *O. proteus* was found only in low- to mid-altitude agro-ecological zones such as the Northern Moist Farmlands, West Nile Farmlands, Central Wooded Savanna, and Southern and Eastern Lake Kyoga Basin. The existence of *O. mutabilis* in all agro-ecologies regardless of altitude suggests its wide adaptability to environmental conditions. However, the higher incidence of *O. mutabilis* in low-altitude agro-ecological zones appears to support the theory that *O. mutabilis* is more abundant in lower-altitude environments [13]. The presence of *O. proteus* in Uganda’s mid-altitude agro-ecological zones is consistent with observations that show that the species is prominent along the Albertine Rift’s mid-altitude section in Kivu [7], implying that the species prefers mid-altitude habitats.

Bean leaf beetles were most common in the Northern Moist Farmlands and least common in the Southwestern Highlands agro-ecological zone (Figure 4). The high prevalence of bean leaf beetles in the Northern Moist Farmlands can be ascribed to the region’s favorable climate. The zone features a bimodal rainfall pattern with the two rainy seasons overlapping [18]. The constant rain from the first season to the second aids in the continuing growth of the bean crop, which may be responsible for the higher beetle density. Furthermore, the Northern Moist Farmlands have a greater diversity of bean leaf beetle hosts than any other agro-ecological zone. For example, there were 41 non-crop potential hosts of bean leaf beetles from the six agro-ecological zones in the current study (Table S10). In the Northern Moist Farmlands, 40 of 41 non-crop potential hosts were found. *Glycine max*, *Hibiscus sabdariffa* (L.), *Vigna radiata* (L.) Wilczek and *V. unguiculata* are more widely grown in this region than in any other region [2]. Among these crops, *V. unguiculata* is the most preferred host of *O. mutabilis* [19].

Bean crops are more prone to beetle damage while they are young, according to our research. *Ootheca* species are seedling pests according to reports [13]. The emergence of bean seedlings, which they eat voraciously to the point of total defoliation, is said to accelerate their field appearance [10].

In the Southern and Eastern Lake Kyoga Basin agro-ecological zone, bean monocrops had a much higher bean leaf beetle density than intercrops; however, no significant differences were seen in other agro-ecological zones. This could be due to the varied agro-ecologies’ cropping system’s complexity. Beans are intercropped with practically every crop in Uganda at varying ratios and planting dates, which may affect the occurrence of bean leaf beetles. Pest occurrence in intercrops is influenced by cultivation strategies such as crop ratios and relative planting dates [20]. Pest incidence is higher in bean monocrops than in intercrops, according to other studies [21]. Low bean leaf beetle occurrences associated with intercropping show that the strategy could help in reducing bean leaf beetle populations. However, studies should be performed to better understand the effects of intercropping when crop type, crop ratios and relative planting dates are taken into account.

In this study, some Chrysomelidae beetles that were found on beans are known key pests of other plant species. For example, *Nisotra* species are major pests of okra *Abelmoschus esculentus* (L.) Moench [22], while *Lamprocopa* species are pests of pumpkin *Cucurbita maxima* Duchesne [23]. Studies to determine the damage–yield relationships of the recovered bean leaf beetle species will be vital in understanding their importance as common bean pests in Uganda.

## 5. Conclusions

Three *Ootheca* species (*O. mutabilis, O. proteus* and *O. orientalis*) were recovered on bean plants in Uganda. The most common species, *O. mutabilis,* was identified in all of the agro-ecological zones studied. Bean leaf beetles are most highly infested in the Northern Moist Farmlands although they cause significant damage in other zones. High bean leaf beetle numbers and damage are linked to the first season rains. Furthermore, our research found that the bean crop is more vulnerable to bean leaf beetle damage during its early phases of development.

Our study provides a foundation for assessing the importance of *Ootheca* species as common bean pests in Uganda. Furthermore, our findings will aid in the development of appropriate bean leaf beetle management approaches in Uganda.

Future research should be conducted to ascertain whether the non-crop potential hosts in this study are actually hosts of bean leaf beetles, especially the *Ootheca* species.

## Figures and Tables

**Table 1:** Districts sampled in different agro-ecological zones during 2016 and 2017 rains in Uganda.

Season	Agro-Ecological Zone					
	NMF	WNF	CWS	WFL	SWH	SEK
2016 first rains	Lira, Oyam, Dokolo	Arua, Yumbe, Maracha	Nakasongola, Mityana, Rakai	Hoima, Bulisa, Masindi	Kabale, Kisoro, Kanungu	Serere, Palisa, Amuria
2016 second rains	Lira, Oyam, Apac	Zombo, Yumbe, Arua	Lwengo, Rakai, Nakasongola	Hoima, Bulisa, Hoima	Kabale, Kisoro, Rubirizi/Bushenyi	Serere, Amuria, Kumi
2017 first rains	Gulu, Amuru, Nwoya	Zombo, Maracha, Arua	Lwengo, Mityana, Nakasongola	Hoima, Bulisa, Masindi	Kabale, Kisoro, Kanungu	Kumi, Amuria, Mbale

Key: NMF = Northern Moist Farmlands, WNF = West Nile Farmlands, CWS = Central Wooded Savanna, WFL = Western Mid-Altitude Farmlands and the Semliki Flats, SWH = Southwestern Highlands, SEK = Southern and Eastern Lake Kyoga Basin.

**Table 2:** Relative abundance (%) of leaf beetles in Ugandan agro-ecological zones from 2016 to 2017.

Species	Agro-Ecological Zone						Mean
	NMF	WNF	CWS	WFL	SWH	SEK	
*Ootheca mutabilis*	58.3	45.5	79.2	94.3	45.8	98.6	70.3
Alticinae sp.	0.3	0	0	0	48.2	0	8.1
*Ootheca proteus*	21.9	12.9	4.4	0	0	1.4	6.7
*Nisotra* spp.	7.3	20.9	0	2.9	0	0	5.2
*Lema* spp.	9.3	13.1	2.2	0.3	0	0	4.2
*Diacantha* spp.	0.5	1.5	0.8	0.3	6	0	1.5
Others	2.4	6.1	13.4	2.0	0	0	4.0

Key: NMF = Northern Moist Farmlands, WNF = West Nile Farmlands, CWS = Central Wooded Savanna, WFL = Western Mid-Altitude Farmlands and the Semliki Flats, SWH = Southwestern Highlands, SEK = Southern and Eastern Lake Kyoga Basin.

## Data Availability

All data are provided in the main body of the published article and Supplementary Material.

## Supplementary Materials

The following are available online at https://www.mdpi.com/article/10.3390/insects12111048/s1, Table S1: Weather variables of the sampled agro-ecological zones during the sampling period, Table S2: The breakdown of genera or species categorized under others in Table 2, Table S3: Foliar damage due to bean leaf beetles across seasons, Table S4: Influence of different bean growth stages on bean leaf beetle populations in Uganda, Table S5: Influence of cropping systems on foliar damage in different agro-ecological zones in Uganda, Table S6: Influence of host plant density on bean leaf beetle populations and foliar damage across agro-ecological zones, Table S7: Effect of time of sampling on occurrence of bean leaf beetles, Table S8: Influence of crop history on bean leaf beetle populations and foliar damage across agro-ecological zones, Table S9: Influence of insecticide use history on bean leaf beetle populations and foliar damage across agro-ecological zones, Table S10: Potential non-crop hosts of bean leaf beetles and their host agro-ecological zones.
