# Supplementary material for: Distribution and Relative Abundance of Bean Leaf Beetles (Ootheca spp.) (Insecta: Coleoptera: Chrysomelidae) in Uganda
Source: Insects. 2021 Nov 22;12(11):1048. doi: 10.3390/insects12111048 (PMC8623429; doi:10.3390/insects12111048)
Supplement: Supplementary file 1 [file insects-12-01048-s001.zip › insects-1398441-supplementary.pdf]

Distribution and Relative Abundance of Bean Leaf Beetles (*Ootheca* spp.) (Insecta: Coleoptera: Chrysomelidae) in Uganda

Charles Halerimana[1,2], Samuel Kyamanywa[1], Samuel Olaboro[3], Pamela Paparu[3], Stanley T. Nkalubo[3], John Colvin[4], Robert A. Cheke[4], Thomas Wagner[5], Susan E. Seal[4], Darren J. Kriticos[6,7] and Michael H. Otim[3,*]

[1]Department of Agricultural production, College of Agricultural and Environmental Sciences, Makerere University, P.O.Box, 7062, Kampala, Uganda, chahalerimana@gmail.com (C.H); skyamanywa@gmail.com (S.K)

[2]National Coffee Research Institute, P.O.Box, 185, Mukono, Uganda, chahalerimana@gmail.com (C.H)

[3] National Crops Resources Research Institute, P.O.Box, 7084, Kampala, Uganda, motim9405@gmail.com (M.H.O); pamela.paparu@gmail.com (P.P); tamusange@gmail.com (S.T.N); olasamuel94@gmail.com(S.O)

[4] Natural Resources Institute, University of Greenwich at Medway, Central Avenue, Chatham Maritime, Kent ME4 4TB, United Kingdom, S.E.Seal@greenwich.ac.uk (S.E.S); J.Colvin@greenwich.ac.uk (J.C); R.A.Cheke@greenwich.ac.uk (R.A.C)

[5] Universität Koblenz-Landau, Institut für Integrierte Naturwissenschaften – Biologie, Universitätstrasse 1, 56070 Koblenz, Germany, thwagner@uni-koblenz.de (T.W)

[6] Commonwealth Scientific and Industrial Research Organization GPO 1700, Canberra, 2601, Australia, Darren.Kriticos@csiro.au (D.J.K)

[7] School of Biology, University of Queensland, 4072 Brisbane, Australia, Darren.Kriticos@csiro.au (D.J.K)

Corresponding author. Michael H. Otim

National Agricultural Research Organization

National Crops Resources Research Institute (NaCRRI) Namulonge

P.O. Box, 7084, Kampala, Uganda

Telephone: +256772897040

Email: motim9405@gmail.com

Table S1. Weather variables of the sampled agro-ecological zones during the sampling period [18].

Season	Month	Agro-ecological zones																	
		Northern Moist Farmlands			West Nile Farmlands			Central Wooded Savanna			Western Mid-Altitude Farmlands and the Semliki Flats			Southern and Eastern Lake Kyoga Basin			Southwestern Highlands		
		Temp (^{O}C)	R/H (%)	R/F (mm)	Temp (^{O}C)	R/H (%)	R/F (mm)	Temp (^{O}C)	R/H (%)	R/F (mm)	Temp (^{O}C)	R/H (%)	R/F (mm)	Temp (^{O}C)	R/H (%)	R/F (mm)	Temp (^{O}C)	R/H (%)	R/F (mm)
2016A	Apr	25.0	71.0	219.7	24.3	67.5	234.0	23.7	72.5	122.2	22.2	85.5	168.8	24.8	65.0	178.3	19.5	85.5	199.0
	May	24.6	70.0	228.7	23.4	69.5	143.0	24.4	72.0	105.5	22.2	76.0	19.6	24.2	71.0	128.7	19.4	80.5	81.0
	Jun	24.5	66.0	92.4	23.0	68.0	68.4	22.8	66.0	61.9	21.6	65.5	18.7	24.2	64.0	63.6	18.0	74.0	33.0
	Mean	24.7	69.0	180.3	23.5	68.3	148.5	23.6	70.2	96.5	22.0	75.7	69.0	24.4	66.7	123.5	18.9	80.0	105.0
2016B	Jul	23.5	71.5	204.3	22.0	74.0	173.7	22.1	71.0	71.1	21.9	66.5	6.2	23.6	67.0	32.3	18.0	68.5	19.0
	Aug	23.9	68.0	72.7	22.1	71.0	71.1	22.9	71.0	87.8	21.9	64.0	7.0	23.7	65.5	100.9	18.4	64.0	43.0
	Sep	23.9	65.0	119.6	22.4	70.5	171.5	22.8	71.5	142.7	21.9	67.0	29.6	24.0	62.0	121.2	19.1	71.5	184.0
	Oct	23.1	60.5	143.4	22.5	67.5	155.9	23.2	68.5	290.8	22.2	71.0	59.0	24.3	60.5	137.5	18.7	76.0	130.0
	Nov	23.7	59.0	59.3	23.0	63.5	98.4	23.7	67.0	101.6	22.0	79.0	112.1	24.4	57.5	134.3	18.3	79.5	124.0
	Dec	24.7	44.0	0.0	23.8	50.5	0.0	24.3	51.5	34.9	21.4	76.5	65.4	25.5	40.5	5.7	18.1	75.5	24.0
	Mean	23.8	61.3	99.9	22.6	66.2	111.8	23.1	66.8	121.5	21.9	70.7	46.6	24.3	58.8	88.7	18.4	72.5	87.0
2017A	Jan	26.7	32.5	3.3	25.0	42.0	1.0	25.3	43.5	6.1	21.1	67.5	52.3	26.3	41.0	18.9	18.7	69.5	41.0
	Feb	26.6	46.0	34.6	25.3	48.0	14.3	25.5	54.0	92.8	23.1	77.5	132.0	26.1	49.5	147.2	18.4	77.0	50.0
	Mar	26.3	62.5	181.8	25.5	54.5	99.7	25.0	59.5	112.5	22.3	79.5	166.8	25.2	60.0	90.4	18.3	80.5	115.0
	Apr	26.4	60.5	151.9	24.2	62.5	129.4	24.8	63.5	113.3	22.0	81.0	154.6	25.1	60.0	155.7	18.1	81.0	72.0
	May	25.1	70.5	138.1	23.4	67.0	128.0	24.1	69.0	130.6	21.8	83.0	93.8	23.4	70.0	150.6	17.9	80.5	76.0
	Jun	25.4	66.5	166.1	22.9	69.0	146.4	23.7	68.5	253.1	20.5	75.5	20.1	23.9	65.5	55.1	18.0	70.0	5.0
	Mean	26.1	56.4	112.6	24.4	57.2	86.5	24.7	59.7	118.1	21.8	77.3	103.3	25.0	57.7	102.9	18.2	76.4	60.0

Key: 2016A = 2016 first rains, 2016B = 2016 second rains, 2017A = 2017 first rains. Temp = temperature, R/F = rainfall, R/H = relative humidity. Temperatures presented are the averages of maximum and minimum temperatures of each month. Relative humidity presented is the average of humidity at 9 am and 3pm for each month.

Table S2. The breakdown of genera or species categorized under others in Table 2

Genera/Species	Agro-ecological zone						Mean
	NMF	WNF	CWS	WFL	SWH	SEK	
Buphonella	0	0	6.1	0	0	0	1.0
Parasbecesta ruwensorica	0.8	1.4	0	0	0	0	0.4
Exosoma pecipenis	0	1.2	0	1.2	0	0	0.4
Neobarombiella	0	0	3.8	0	0	0	0.6
Afrophthalma	0	0	2.6	0.6	0	0	0.5
Chrysochrus	0.6	0.08	0	0	0	0	0.1
Exosoma	0	1.2	0	0.8	0	0	0.3
Lamprocopa	0.06	3.0	0	0.6	0	0	0.6
Plagiodera	0.5	0	0	0	0	0	0.08

Key: NMF = Northern Moist Farmlands, WNF = West Nile Farmlands, CWS = Central Wooded Savanna, WFL = Western Mid-Altitude Farmlands and the Semliki Flats, SWH = Southwestern Highlands, SEK = Southern and Eastern Lake Kyoga Basin

Table S3. Foliar damage due to bean leaf beetles across seasons

Season	Foliar damage per plant (±SE)
2016 first rains	0.87±0.05a
2016 second rains	0.79±0.03a
2017 first rains	0.87±0.04a

Within a column, values bearing the same letter are not significantly different at $p = 0.05$

Table S4. Influence of different bean growth stages and reproductive phases (abbreviations are listed in Figure 7) on bean leaf beetle populations in Uganda

Bean growth stage	Bean leaf beetles per plant (±SE)
V1	0.09±0.02a
V2	0.10±0.03a
V3	0.07±0.02a
V4	0.09±0.02a
V5	0.05±0.02a
R1	0.13±0.03a
R2	0.11±0.03a
R3	0.11±0.03a
R4	0.04±0.02a
R5	0.04±0.01a
R6	0.05±0.02a

Within a column, values bearing the same letter are not significantly different at $p = 0.05$

Table S5. Influence of cropping systems on foliar damage in different agro-ecological zones in Uganda.

Agro-ecological zone	Foliar damage per plant (±SE)	
	**Monocrop**	**Intercrop**
Northern Moist Farmlands	1.12±0.07de	1.19±0.04e
West Nile Farmlands	0.85±0.07bcd	0.95±0.05cde
Central Wooded Savanna	0.80±0.11bcde	0.89±0.06cd
Western Mid-Altitude Farmlands and the Semliki Flats	0.21±0.11a	0.55±0.06ab
Southwestern Highlands	0.52±0.08ab	0.54±0.07ab
Southern and Eastern Lake Kyoga Basin	0.76±0.09bcd	0.69±0.07bc

Within a row, values bearing the same letter or letter combinations are not significantly different at $p = 0.05$

Table S6. Influence of host plant density on bean leaf beetle populations and foliar damage across agro-ecological zones

Agro-ecological zone	Bean leaf beetles per plant (±SE)		Foliar damage per plant (±SE)	
	**Low density**	**High density**	**Low density**	**High density**
Northern Moist Farmlands	0.14±0.01b	0.20±0.06ab	1.15±0.04e	1.40±0.16de
West Nile Farmlands	0.04±0.01a	0.05±0.07ab	0.92±0.04cd	0.90±0.18abcde
Central Wooded Savanna	0.08±0.02ab	0.08±0.12ab	0.87±0.05cd	1.02±0.31abcde
Western Mid-Altitude Farmlands and the Semliki Flats	0.07±0.02ab	0.25±0.17ab	0.46±0.05a	0.75±0.43abcde
Southwestern Highlands	0.02±0.02a	0.00±0.12ab	0.54±0.05ab	0.33±0.30abcde
Southern and Eastern Lake Kyoga Basin	0.09±0.02ab	0.00±0.06ab	0.75±0.05bc	0.43±0.17abc

Within a row, values bearing the same letter or letter combinations are not significantly different at $p = 0.05$ for each category

Table S7. Effect of time of sampling on occurrence of bean leaf beetles

Time of sampling	Bean leaf beetles per plant (±SE)
Early morning hours (8-11 a.m.)	0.06±0.01a
Afternoon hours (12-3 p.m.)	0.09±0.27a
Evening hours (4-5 p.m.)	0.10±0.01a

Within a column, values bearing the same letter are not significantly different at $p = 0.05$

Table S8. Influence of crop history on bean leaf beetle populations and foliar damage across agro-ecological zones

Agro-ecological zone	Bean leaf beetles per plant (±SE)		Foliar damage per plant (±SE)	
	**Host**	**Non-host**	**Host**	**Non-host**
Northern Moist Farmlands	0.14±0.03ab	0.14±0.01b	1.12±0.08de	1.18±0.04e
West Nile Farmlands	0.03±0.04ab	0.04±0.02a	1.12±0.11de	0.88±0.04cd
Central Wooded Savanna	0.05±0.05ab	0.09±0.02ab	0.71±0.12abcd	0.91±0.06cd
Western Mid-Altitude Farmlands and the Semliki Flats	0.03±0.03ab	0.11±0.03ab	0.34±0.08a	0.57±0.08ab
Southwestern Highlands	0.05±0.03ab	0.02±0.02a	0.51±0.09ab	0.55±0.06ab
Southern and Eastern Lake Kyoga Basin	0.06±0.03ab	0.09±0.02ab	0.61±0.09abc	0.78±0.07bcd

Within a row, values bearing the same letter or letter combinations are not significantly different at $p = 0.05$ for each category

Table S9. Influence of insecticide use history on bean leaf beetle populations and foliar damage across agro-ecological zones

Agro-ecological zone	Bean leaf beetles per plant (±SE)		Foliar damage per plant (±SE)	
	**Used**	**Not used**	**Used**	**Not used**
Northern Moist Farmlands	0.10±0.02ab	0.16±0.01b	1.21±0.07ef	1.15±0.04df
West Nile Farmlands	0.04±0.04ab	0.04±0.01a	0.91±0.11bcdef	0.92±0.04ce
Central Wooded Savanna	0.01±0.06ab	0.09±0.02ab	0.60±0.16abcd	0.91±0.06cdef
Western Mid-Altitude Farmlands and the Semliki Flats	0.00±0.07ab	0.08±0.02ab	0.60±0.19abcdef	0.45±0.05a
Southwestern Highlands	0.05±0.06ab	0.02±0.02a	0.67±0.17abcdef	0.52±0.05ab
Southern and Eastern Lake Kyoga Basin	0.08±0.03ab	0.09±0.02ab	0.77±0.09abc	0.69±0.07abc

Within a row, values bearing the same letter or letter combinations are not significantly different at $p = 0.05$ for each category

Table S10. Potential non-crop hosts of bean leaf beetles and their host agro-ecological zones

Number	Species/subspecies name	Family name	Life form	Host agro-ecological zone
1	*Abelmoschus esculentus* (L.) Moench	Malvaceae	herb	NMF, WNF
2	*Abutilon hirtum* (Lam.) Sweet	Malvaceae	herb	NMF, SEK, WNF, CWS, SWH, WFL
3	*Abutilon mauritanum* (Jacq.) Medik.	Malvaceae	herb	NMF, SEK, WNF, CWS, SWH, WFL
4	*Acalypha crenata* Hochst. ex A. Rich.	Euphorbiaceae	herb	NMF
5	*Acanthospermum hispidum* DC.	Asteraceae	herb	NMF
6	*Amaranthus dubius* Mart. ex Thell.	Amaranthaceae	herb	NMF, SEK, WNF, CWS, SWH, WFL
7	*Amaranthus hybridus cruentus* (L.) Thell.	Amaranthaceae	herb	NMF, SEK, WNF, CWS, SWH, WFL
8	*Aspilia kotschyi* (Sch. Bip. ex Hochst.) Oliv.	Asteraceae	herb	NMF, NWF
9	*Asystasia gangetica* (L.) T. Anderson	Acanthaceae	herb	NMF
10	*Asystasia mysorensis* (Roth) T. Anderson	Acanthaceae	herb	NMF, SEK
11	*Celosia trigyna* L.	Amaranthaceae	herb	NMF, SEK, WNF
12	*Clerodendrum umbellatum* Poir.	Verbanaceae	herb	NMF
13	*Combretum collinum* Fresen.	Combretaceae	tree	NMF
14	*Corchorus olitorius* L.	Tiliaceae	herb	NMF, WNF
15	*Crossandra subacaulis* C. B. Clarke	Acanthaceae	herb	NMF, CWS
16	*Crotalaria* sp.	Papilionaceae	herb	NMF, SEK, WFL
17	*Cyathula prostrata* (L.) Blume	Amaranthaceae	herb	NMF, SEK
18	*Desmodium gangeticum* (L.) DC.	Papilionaceae	herb	NMF, WNF
19	*Desmodium tortuosum* (Sw.) DC.	Papilionaceae	herb	NMF, SEK, WNF
20	*Dombeya burgessiae* Gerrard ex Harv.	Sterculiaceae	shrub	WFL
21	*Dyschoriste nagchana* (Nees) Bennet	Acanthaceae	herb	NMF, SEK, WFL
22	*Erlangea cordifolia* (Benth. ex Oliv.) S. Moore	Asteraceae	herb	NMF
23	*Grewia mollis* Juss.	Tiliaceae	shrub	NMF
24	*Hibiscus acetosella* Welw. ex Hiern.	Malvaceae	herb	NMF, SEK, WNF
25	*Abelmoschus moschatus* Medik.	Malvaceae	herb	NMF, WNF
26	*Kosteletzkya adoensis* (Hochst. ex A. Rich.) Mast.	Malvaceae	herb	NMF, SEK, SWH
27	*Leonotis nepetifolia* (L.) R. Br.	Lamiaceae	herb	NMF
28	*Lipia abyssinica* (Otto & A. Dietr.) Cufod.	Verbenaceae	herb	NMF, SEK, WNF, CWS
29	*Lippia woodii* Mold.	Verbenaceae	herb	NMF

Table S10 *Cont.*				
**Number**	**Species/subspecies name**	**Family name**	**Life form**	**Host agro-ecological zone**
30	*Micrococca merculiaris* (L.) Benth.	Euphorbiaceae	herb	NMF
31	*Oxygonum sinuatum* (Hochst. & Steud. ex Meisn.) Dammer	Polygonaceae	herb	NMF
32	*Philenoptera laxiflora* (Guill. & Perr.) Roberty	Papilionaceae	tree	NMF
33	*Senna obtusifolia* (L.) H. S. Irwin & Barneby	Caesalipinaceae	herb	NMF
34	*Sida alba* L.	Malvaceae	herb	NMF, SEK, WNF, CWS, SWH, WFL
35	*Sida rhombifolia* L.	Malvaceae	herb	NMF, SEK, WNF, CWS, SWH, WFL
36	*Synerdrella nodiflora* L. (Gaertn.)	Asteraceae	herb	NMF, SEK
37	*Trichodesma zeylanicum* (Burm. f.) R. Br.	Boraginaceae	herb	NMF
38	*Triumfetta macrophylla* K. Schum.	Tiliaceae	herb	NMF, SEK, WNF, CWS, SWH, WFL
39	*Triumfetta rhomboidea* Jacq.	Tiliaceae	herb	NMF, SEK, WNF, CWS, SWH, WFL
40	*Urena lobata* L.	Malvaceae	herb	NMF, SEK, WNF, CWS, SWH, WFL
41	*Vernonia cinerea* (L.) Less.	Asteraceae	herb	NMF, SEK

Key: NMF = Northern Moist Farmlands, WNF = West Nile Farmlands, CWS = Central Wooded Savanna, WFL = Western Mid-Altitude Farmlands and the Semliki Flats, SWH = Southwestern Highlands, SEK = Southern and Eastern Lake Kyoga Basin
